# An ontology-based modelling system (OBMS) for representing behaviour change theories applied to 76 theories

**DOI:** 10.12688/wellcomeopenres.16121.1

**Published:** 2020-07-23

**Authors:** Joanna Hale, Janna Hastings, Robert West, Carmen E. Lefevre, Artur Direito, Lauren Connell Bohlen, Cristina Godinho, Niall Anderson, Silje Zink, Hilary Groarke, Susan Michie

**Affiliations:** 1 Centre for Behaviour Change, University College London, London, UK; 2 Research Department of Epidemiology & Public Health, University College London, London, UK; 3 Department of Behavioral and Social Sciences, Brown University School of Public Health, Providence, Rhode Island, USA; 4 Católica Research Centre for Psychological, Family and Social Wellbeing, Universidade Católica Portuguesa, Lisboa, Portugal; 5 Center for Research and Social Intervention, Instituto Universitário de Lisboa, Lisboa, Portugal; 6 NIHR Health Protection Research Unit in Behavioural Science and Evaluation, University of Bristol, Bristol, UK

**Keywords:** behaviour, behaviour change, theory, ontology, modelling, theoretical synthesis

## Abstract

**Background:** To efficiently search, compare, test and integrate behaviour change theories, they need to be specified in a way that is clear, consistent and computable. An ontology-based modelling system (OBMS) has previously been shown to be able to represent five commonly used theories in this way. We aimed to assess whether the OBMS could be applied more widely and to create a database of behaviour change theories, their constructs and propositions.

**Methods:** We labelled the constructs within 71 theories and used the OBMS to represent the relationships between the constructs. Diagrams of each theory were sent to authors or experts for feedback and amendment. The 71 finalised diagrams plus the five previously generated diagrams were used to create a searchable database of 76 theories in the form of construct-relationship-construct triples. We conducted a set of illustrative analyses to characterise theories in the database.

**Results:** All 71 theories could be satisfactorily represented using this system. In total, 35 (49%) were finalised with no or very minor amendment. The remaining 36 (51%) were finalised after changes to the constructs (seven theories), relationships between constructs (15 theories) or both (14 theories) following author/expert feedback. The mean number of constructs per theory was 20 (min. = 6, max. = 72), with the mean number of triples per theory 31 (min. = 7, max. = 89). Fourteen distinct relationship types were used, of which the most commonly used was ‘influences’, followed by ‘part of’.

**Conclusions:** The OBMS can represent a wide array of behavioural theories in a precise, computable format. This system should provide a basis for better integration and synthesis of theories than has hitherto been possible.

## Introduction

Many of society’s pressing global problems, such as environmental degradation, social conflict and poor health, require changes in a wide range of human behaviours at individual, community and population levels. Interventions are likely to be more effective if they are informed by behaviour change theories which are developed to represent a body of knowledge and understanding about processes of change ( Craig *et al.*, 2008; Wolfenden *et al.*, 2018; Gourlan *et al.*, 2016). However, many interventions aimed at changing behaviour are not explicitly guided by theory ( Michie & Prestwich, 2010; Prestwich *et al.*, 2014). We define theory as ‘a set of concepts and/or statements with specification of how phenomena relate to each other. Theory provides an organising description of a system that accounts for what is known, and explains and predicts phenomena’, ( Michie *et al.*, 2005; qeios.com/read/definition/631).

Those wishing to draw on theory in developing interventions are faced with a plethora of different theories and potential underlying mechanisms through which change can occur. A systematic review of 83 theories of behaviour change identified several barriers to effective theory use in intervention design and evaluation ( Davis *et al.*, 2015). Theories tend to overlap in terms of content and scope, often lack detail in terms of clear definitions of constructs and relationships, and most include only a subset of relevant constructs within their stated scope. Different labels are also used interchangeably to describe the same construct, or the same labels are used to describe different constructs ( Michie *et al.*, 2014).

Theories of behaviour are generally represented through natural language, sometimes with visualisations that may be whole, simplified or partial. Whilst natural language benefits from the richness and subtlety of language, it can introduce ambiguity and limits comparison, integration and testing, holding back the advancement of understanding human behaviour and hence the potential to develop effective interventions ( Fried, 2020; Muthukrishna & Henrich, 2019; Oberauer & Lewandowsky, 2019). Moreover, it is often unclear whether natural language descriptions of theories written by authors are intended to be testable propositions.

To make theories more useable and useful, we need a method for accurately representing them that can identify ambiguities and allow comparisons in terms of content, scope, and predictions. Such a method would enable theory integration and development by making propositions within existing theories more easily discoverable, and facilitate the testing (including falsification) of propositions by making them more precise. More precise and accurate theory representations may also enhance the selection and application of theories when developing and evaluating interventions for real-world problems. Several methodologies have been developed to improve openness, precision and rigour in the cycle of psychological theory development (e.g. Borsboom *et al.*, 2020; Forstmann *et al.*, 2011; Guest & Martin, 2020; Haslbeck *et al.*, 2019; van Rooij & Baggio, 2020). These methodologies agree that theories should be formally specified in unambiguous terms, for example as a set of equations or computational model. A systematic method capable of formally representing any kind of theory proposition in a way that can be consistently interpreted, whether read verbally, computationally or diagrammatically, can improve theory development and synthesis. An additional advantage of such a system is that theories can be made ‘computer-readable’ to enable searching and other computer-assisted operations.

One such method, termed an ‘ontology-based modelling system’ (OBMS) has been developed to enable systematic and precise theory representation ( West *et al.*, 2019). The OBMS is a system for modelling theories of behaviour and behaviour change, using a formal representation of the constructs within theories and the ways in which constructs may relate to or interact with each other. Ontologies are formal representations of entities and relations in a given domain, in which each entity is assigned an unambiguous label and a clear definition. The OBMS is not an ontology (as ontologies typically seek to represent knowledge about the world, rather than theories about it) but uses an equivalent modelling system to that used in ontologies to express theories formally as a set of construct-relationship-construct triples, such as "intention-influences-behaviour" or "anxiety-is correlated with-performance". The OBMS also provides a system for graphically representing different types of triples, which allows a given theory to be represented as a diagram that fully specifies all the proposed relationships among its constructs in an unambiguous format. The viability of the OBMS was demonstrated by West *et al.* (2019) by successfully capturing the propositions of five commonly used behaviour change theories ( Painter *et al.*, 2008; Prestwich *et al.*, 2014): the Health Belief Model (HBM; Rosenstock, 1974), the Information-Motivation-Behavioural Skill Model ( Fisher & Fisher, 1992), Social Cognitive Theory ( Bandura, 1986), the Theory of Planned Behaviour ( Ajzen, 1991), and the Transtheoretical Model ( DiClemente *et al.*, 1991).

**Figure 1.**
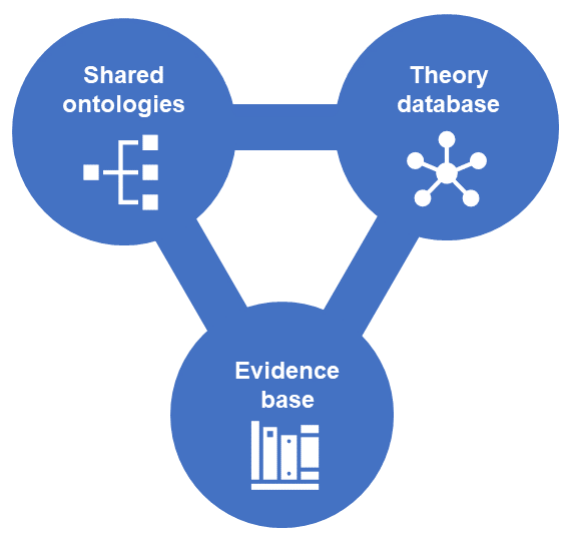
Combined use of shared ontologies, theory database and evidence base. Shared ontologies provide an agreed and coherent system of reference, against which we can map a database of theories that have been specified using the OBMS. Both can be annotated with evidence for the links proposed.

The application of the OBMS to a much wider range of existing and future theories can facilitate theory testing and development, particularly when used in conjunction with ontological tools. Ontological tools provide an agreed and coherent system of reference, against which we can map theories that have been formally specified, and annotate evidence for the links proposed ( Figure 1). In this way, theory propositions can be shared, tested and evidence synthesised to advance theory much more efficiently. One specific ontological tool was developed in The Human Behaviour Change Project, a wide programme of research which uses behavioural science and machine learning to answer ‘What intervention(s) work, compared with what, how well, with what exposure, with what behaviours, for how long, for whom, in what settings and why?’ ( Michie *et al.*, 2020a). This work involves the development of an overarching Behaviour Change Intervention Ontology (BCIO) which specifies entities making up an intervention scenario and how they are related ( Michie *et al.*, 2020b). A recent scoping review informing the development of the BCIO identified 15 existing ontologies related to human behaviour change, but none that represented the breadth and details of human behaviour change ( Norris *et al.*, 2019). Therefore, the BCIO provides an ontology that can be used to systematise the reporting of behaviour change interventions, make evidence synthesis more effective and point to what is not known. The BCIO builds on the Behaviour Change Technique Taxonomyv1 ( Michie *et al.*, 2013) and a subsequent programme of work linking behaviour change techniques with frequently occurring mechanisms of action and generating a Theory and Techniques Tool for easy identification of these links ( theoryandtechniquetool.humanbehaviourchange.org). The research generated links between 61 behaviour change techniques and 26 mechanisms of action, based on authors’ reports in published behaviour change literature ( Carey *et al.*, 2019), expert consensus ( Connell *et al.*, 2019) and the triangulation of the two sources of evidence ( Johnston *et al.*, 2018).

The present study aimed to (i) investigate whether a wide range of behaviour change theories could be represented using the OBMS with sufficient precision to capture all constructs and relationships in computer-readable diagrammatic form and (ii) build a database of theories represented using this system that can be searched for construct names and relationships. This type of structured database provides a basis for logical and mathematical inferences. In the present study, we conducted a set of illustrative analyses to characterise theories in the database, with further research currently underway to use computational methods to identify ‘canonical’ theories.

## Methods

### Selection of theories

**Figure 2.**
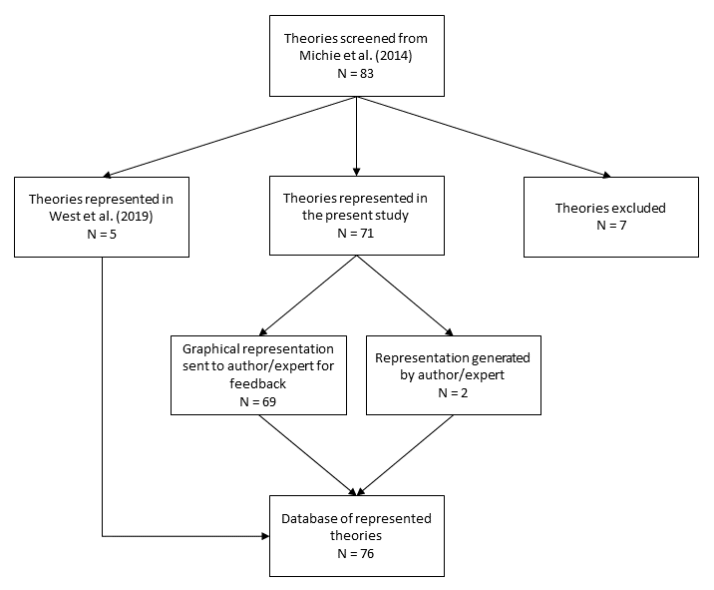
Flowchart of theories screened and included in the present study.

We screened descriptions of 83 theories identified in a multidisciplinary review of 83 theories of behaviour and behaviour change ( Davis *et al.*, 2015; Michie *et al.*, 2014) for inclusion in this study ( Figure 2). Theories were numbered 1–83 in alphabetical order for ease of identification throughout the study (see *Extended data* for theory numbers; osf.io/dcqft ( Hale *et al.*, 2020)). In total 71 theories were included for representation in the present study with five theories already represented by West *et al.* (2019). All 76 theories were included in the final database. Seven theories were excluded according to the following criteria:

1. Did not clearly specify at least one relationship linking a construct to behaviour or behaviour change. Two theories did not meet this criterion: Goal Directed Theory ( Bagozzi, 1992), and Social Action Theory ( Weber, 1978) both contain constructs that describe behaviour, i.e. goal achievement and social action, respectively. However, neither describes a link between behaviour and one of the other constructs in the theory.
2. Did not include behaviour or a similar construct. Following correspondence with theory experts, Classical Conditioning Theory ( Pavlov, 1927) was deemed ineligible as it focuses on autonomic processes and emotional responses.
3. Had been explicitly integrated into a more comprehensive theory by the same authors. Four theories were excluded on this basis: the Extended Information Processing Model ( Flay *et al.*, 1980), the Integrative Models of Factors influencing Smoking ( Flay *et al.*, 1983), the Integrative Model of Health Attitude and Behaviour Change ( Flay, 1981), and the Integrative Model of Factors influencing Smoking, Health Attitude and Behaviour Change ( Flay *et al.*, 1983) had been incorporated into one more comprehensive, integrative theory, the Theory of Triadic Influence ( Flay & Petraitis, 1994), which was included.

These criteria were decided through discussion between the researchers and study leads (SM and RW).

### Identification of theory constructs

Theory constructs were identified from the original theory sources, using the following definition of a construct: “a component of that model or theory that is a representation of an object, event, state of affairs, feature of one of these, derived from observation and inference” ( Michie *et al.*, 2014, p. 39). At least two researchers independently identified constructs that could be unambiguously derived from theory descriptions. Discrepancies between researchers were resolved through discussion, and if necessary through consultation with the study leads (SM and RW). A further check for accuracy was conducted by comparing constructs extracted from the original sources with those in the theory summaries in Michie *et al.* (2014). Where the new reading of the original theory source led to an omission or addition of a construct compared with Michie *et al.* (2014), the differences were recorded and checked by a further researcher, and were checked with the theory author/expert when sending the theory diagram for feedback (see below section, ‘Author/expert feedback on diagrams’).

### Generation of theory diagrams using the OBMS

At the same time as extracting theory constructs, one researcher drafted an initial diagram of the theory using the OBMS ( West *et al.*, 2019). The diagram was generated using Lucid Chart diagram software ( www.lucidchart.com), which allows constructs (visualised as shapes) to be linked together by labelled relationships (visualised as lines or arrows). Draw.io is a free alternative with similar functionality. Nineteen conceivable types of relationship were initially identified in the development of the OBMS and different symbols and labels were developed to denote each type (see *Extended data* for guide to relationship types; osf.io/8fvdb ( Hale *et al.*, 2020)). The number, wording and visual representation of these relationship types were revised for comprehensiveness and parsimony during the project, resulting in 1 additional relationship type to the types described in West *et al.* (2019). The second and/or third researcher agreed, refined or changed the initial representation in line with their reading of the theory and discussion with the other researcher/s. The agreed diagram was then sent to the theory author or an expert for feedback (see below section, ‘Author/expert feedback on diagrams’). The diagrams of two theories, CEOS ( Borland, 2014) and PRIME Theory ( West & Brown, 2013), were initially drawn by the research team, but early feedback resulted in completely new diagrams being drawn by the theory authors, which did not subsequently need to go through the feedback process.

### Author/expert feedback on diagrams

Theory authors were identified from the original theory sources ( Michie *et al.*, 2014). When contact details were not provided in the theory source, the website of an author’s affiliated institution was consulted. For the 15 authors with whom contact could not be made (14 deceased, 1 retired), alternative theory experts were identified by searching the Web of Science and Scopus databases for authors who had cited the theory most frequently, and then examining published articles by these authors for relevance ( West *et al.*, 2019).

Theory authors and experts were sent a PDF copy of the OBMS diagram of their theory, a guide to the symbols and labels we used to specify the propositions ( *Extended data:* osf.io/8fvdb ( Hale *et al.*, 2020)), and a copy of the published theory review ( Davis *et al.*, 2015) which lists the constructs of each behaviour change theory. They were asked to review the OBMS representation of their theory for its accuracy, including any added or omitted constructs (see section above, ‘Identification of theory constructs’). If no response was received, two reminder emails were sent. In the final reminder, we indicated that we would take a non-response as an indication of approval of our theory representation.

### Revision of diagrams following author/expert feedback

If a theory author/expert responded, we coded the content of their response according to whether it contained the following types of feedback: (1) specific feedback on constructs, relationships and/or structure; (2) general feedback on diagram as a whole; (3) general feedback on process/methodology; (4) reference to readings; (5) queries about diagram; (6) sent revised diagrams; (7) constraints on ability to comment.

A researcher identified necessary changes to the diagram from the content of the feedback and any additional readings sent. A second researcher reviewed this process for each theory. Disagreements were resolved through discussion. The agreed changes were coded into three categories: (1) add/remove/amend constructs; (2) add/remove/amend relationships between constructs; (3) both. Very minor changes such as spelling corrections or hyphenations were not coded. The revised representation was sent back to the author/expert; this process was repeated until a final representation was agreed or until no more response was received. Following each revision of the theory, up to two reminder emails were sent, with the final reminder indicating that we would take a non-response as an indication of approval.

### Generating a searchable database of theories represented using the OBMS

To generate a searchable database of theories, we pooled the five theory representations generated by West *et al.* (2019) and the 71 theory representations generated in the present study. Taking the finalised diagrams of all 76 theories, we derived a formal representation of each theory, i.e. a set of construct-relationship-construct triples. This was done by exporting CSV data from the LucidChart diagram of each theory using the LucidChart process diagram CSV export facility, then running a script in Python (Version 3.8) to identify construct-relationship-construct triples from the CSV data. Where theories contained a construct that was related to another relationship (rather than related to a construct), this was represented by ‘reifying’ (i.e. treating a triple as if it were a construct) the second relationship triple, e.g., “Feedback-influences-[the 'Goals' to 'Persistence' Influences (*) relationship]”. A web interface for the theory database was also implemented in Python, using the Flask web framework (Version 1.1) with search functionality provided by the Whoosh indexing library (Version 2.7). To aid the readability of theories in the database, we generated new diagrams with simpler visual conventions than those used in the theory diagrams sent to authors, e.g. using text labels instead of shapes to denote the types of relationship. The new diagrams were automatically generated from the set of triples for each theory using Python’s library interface to the GraphViz application ( Ellson *et al.*, 2003).

In the present study, descriptive analyses were carried out on the database to explore the mean number of constructs and relationships in each theory and the frequencies of constructs and relationships across theories. A ‘network neighbourhood’ map was constructed to illustrate links to and from two exemplary constructs across all theories, and a bi-clustered percentage containment heat map was constructed to explore the similarities among theories’ constructs across the whole dataset. Both maps were created using Python (Version 3.8).

## Results

### Representation of 71 theories using the OBMS in computer-readable diagrammatic form

All 71 included theories of behaviour could be represented using the OBMS. Table 1 shows how many rounds of revision were required before the theory representations were finalised. It also shows the types of feedback received in each round and the category of changes made to the theories. Of the 69 authors/experts contacted, 50 (72.5%) responded and 38 of those who responded (55.1%) included feedback on the theory representation in their response. The finalised diagrams of the 76 theories represented using the OBMS, including the five theories previously described in West *et al.* (2019) are available in *Extended data* ( osf.io/4urjc/files ( Hale *et al.*, 2020)).

**Table 1.** Summary of the feedback and revision process for 69 theories sent to authors/experts.

Round of revision	Theories finalised (N)	Feedback received (N theories)	Types of feedback received ^a^ (N theories)							Category of changes made (N theories)		
			1	2	3	4	5	6	7	Constructs	Relationships	Both
0 (no changes)	33	2 ^b^	0	2	1	1	0	0	1	--	--	--
1	26	36	32	19	7	15	5	5	1	7	15	14
2	8	12 ^c^	10	6	4	3	2	1	1	1	3	6
3	1	3 ^d^	2	1	0	2	0	0	0	0	1	1
4	1	1	1	0	0	0	0	0	0	0	1	0
All rounds	69	38	34	23	11	17	6	5	3	7	15	14

*Notes:*

^a^(1) specific feedback on constructs, relationships and/or structure; (2) general feedback on diagram as a whole; (3) general feedback on process/methodology; (4) reference to readings; (5) queries about diagram; (6) sent revised diagrams; (7) constraints on ability to comment.

^b^For two theories (Needs-Opportunities-Abilities Model and Social Identity Theory) first round feedback was received but necessary changes were not identified and therefore these theories were finalised with no revision.

^c^For two theories (Feedback Intervention Theory and Self-Regulation Theory) second round feedback was received but necessary changes were not identified and therefore these theories were finalised after one round of revision.

^d^For one theory (Control Theory) third round feedback was received but necessary changes were not identified and therefore this theory was finalised after two rounds of revision.

### A searchable database of 76 OBMS-represented behaviour change theories

The online database of behaviour change theories is accessible at humanbehaviourchange.org/theory-database. The web interface provides online searching and browsing functionality for the database of triples associated with the theories. This provided a formal, computer-readable and searchable database of the 76 theories that have so far been represented using the OBMS.

The database can be searched by the name of any construct in any theory by entering a search string in the search box on the home page. Searches may include wildcards, for example, searching for ‘act*’ will retrieve results related to ‘act’, to ‘actor’ and to ‘action’. If the search retrieves any results, the search results page will display the construct name that was found as well as the theories that that construct was found in. Some constructs are found in multiple theories. For example, the construct ‘action’ occurs in theories ‘Health Action Process Approach’ and ‘Six staged model of communication effects.’

Alternatively, it is possible to browse the database by theory. A full list of the theories currently included in the database is listed at the bottom of the home page. Clicking the link with the name of a theory (whether in the search results or the browse facility) will open a theory page in the theory database. The theory page lists all the triples included in that theory and includes an automatically generated diagram of the relationships specified by the triples.

**Figure 3.**
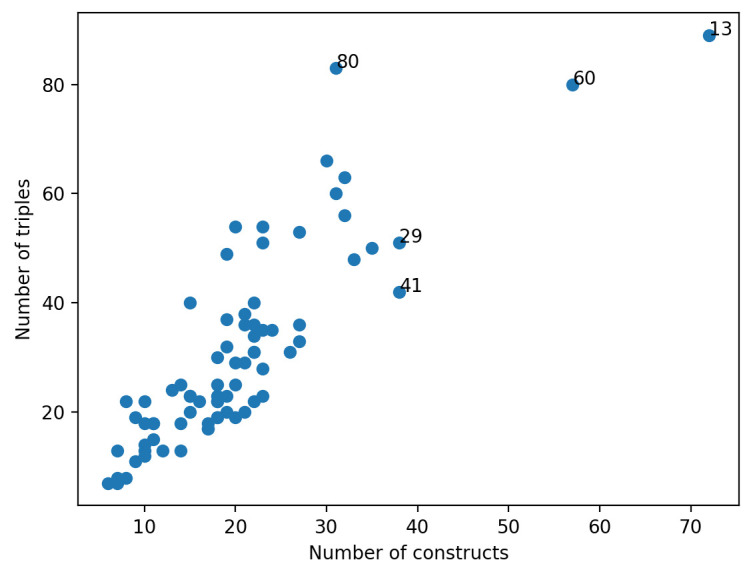
Number of constructs and number of triples per theory. Each dot in the Figure represents a single theory from the theory database. The numbered theories refer to those with a high number of constructs and/or triples. They are: (13) Diffusion of Innovations; (41) Needs-Opportunities-Abilities Model; (60) Social Action Theory (Ewart); and (80) Theory of Triadic Influence.

It was possible to derive several statistics and observations from the database of 76 theories, which were numbered for ease of identification (see *Extended data* for theory numbers; osf.io/dcqft ( Hale *et al.*, 2020)). The mean number of constructs per theory was 20 (min. = 6, max. = 72), while the mean number of triples (i.e. relationships between constructs) per theory was 31 (min. = 7, max. = 89). Broadly, these two counts are correlated, but some theories are denser in connections than constructs and others are denser in constructs ( Figure 3). For example, the five large theories (labelled by number in Figure 3) are theories number 13, 41, 60 and 80 (i.e., “Diffusion of Innovations” theory, “Needs-Opportunities-Abilities Model”, “Social Action Theory (Ewart)” and “Theory of Triadic Influence”, respectively). Amongst these, however, “Social Action Theory (Ewart)” (theory 41) has fewer triples relative to the number of constructs and 80 has fewer constructs relative to the number of triples. As would be expected, most theories have more triples than constructs.

**Figure 4.**
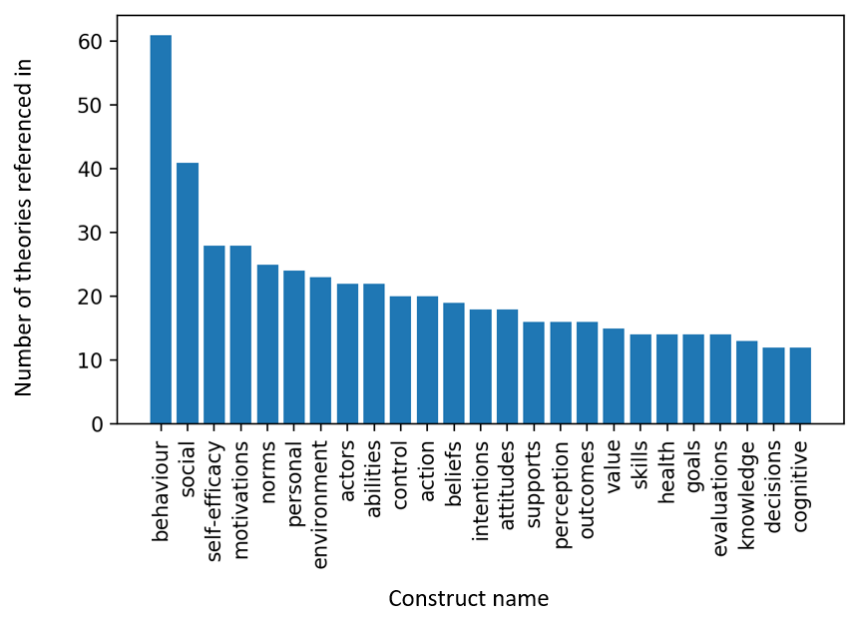
Twenty-five most commonly referenced theory construct names. Bars show how many theories referenced each construct name, either by exact match or within a longer construct name. The calculation of constructs appearing in theories was done using the “lemmatized” form of the construct label so as to combine singular forms with plurals.

In order to see how often each construct in the whole dataset was referenced across theories, we took each construct name (a total of 1,290) and searched for all theories where it matched all or part of a construct name. With this method, if we search for the construct ‘behaviour’, partial matches with ‘coping behaviour’ and ‘attitude to the behaviour’ would be included. We chose to include partial matches in the search because there were very few exact matches of construct names across theories. We also used the “lemmatized” form of the construct label so as to combine singular forms with plurals. ** Using this search method, we found that 1,028 constructs only appeared in one theory. Even with partial matching, only 20% (n = 262) of the constructs appeared in more than one theory. Among the 262 construct names that did get referenced in multiple theories, Figure 4 illustrates the 25 most common.

As we might expect, ‘behaviour’ is the most common construct across all theories, named in 61 theories. The list also includes other crucial constructs that feature commonly in theories of behaviour change, including ‘self-efficacy’, ‘motivation’, ‘ability’, ‘intentions’ and ‘skills’.

Fourteen relationship types were used across the whole dataset of theories. Table 2 lists the counts of relationships used in terms of both the number of theories that a given relation appears in, and the number of triples it is used in across all theories. Thirteen of these relationship types were identified within the five theories represented by West *et al.* (2019); the ‘transitions to’ relationship was added during the present study.

**Table 2.** Relationship counts by relationship type.

Relationship name	Number of theories used in	Total number of triples
Influences	73	995
Part of	56	578
Type of	48	394
Influences (*) [multiplicative]	32	156
Positively influences	29	132
Negatively influences	17	26
Transitions to	15	50
Correlates with	7	18
May influence	1	4
Influences (+) [additive]	1	3
Has attribute	1	1
Value of	1	5
Has start	1	4
Has end	1	4

**Figure 5.**
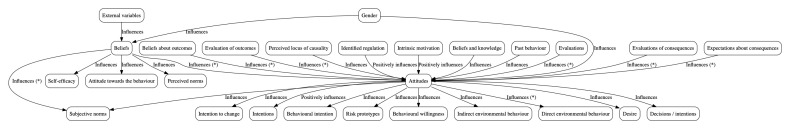
Illustration of relationships to and from constructs, across theories. This diagram shows the ‘influences’ relationships to and from constructs 'beliefs' and 'attitudes', illustrating both shared and distinct factors.

The theory database provides the ability to get an overview across multiple theories. For example, we can probe the ‘network neighbourhood’ of a construct across multiple theories that it appears in to see which relationships have been captured for that construct regardless of which theory they were captured in. For example, Figure 5 illustrates all the ‘influences’ relationships to and from the ‘beliefs’ and ‘attitudes’ constructs across all theories.

**Figure 6.**
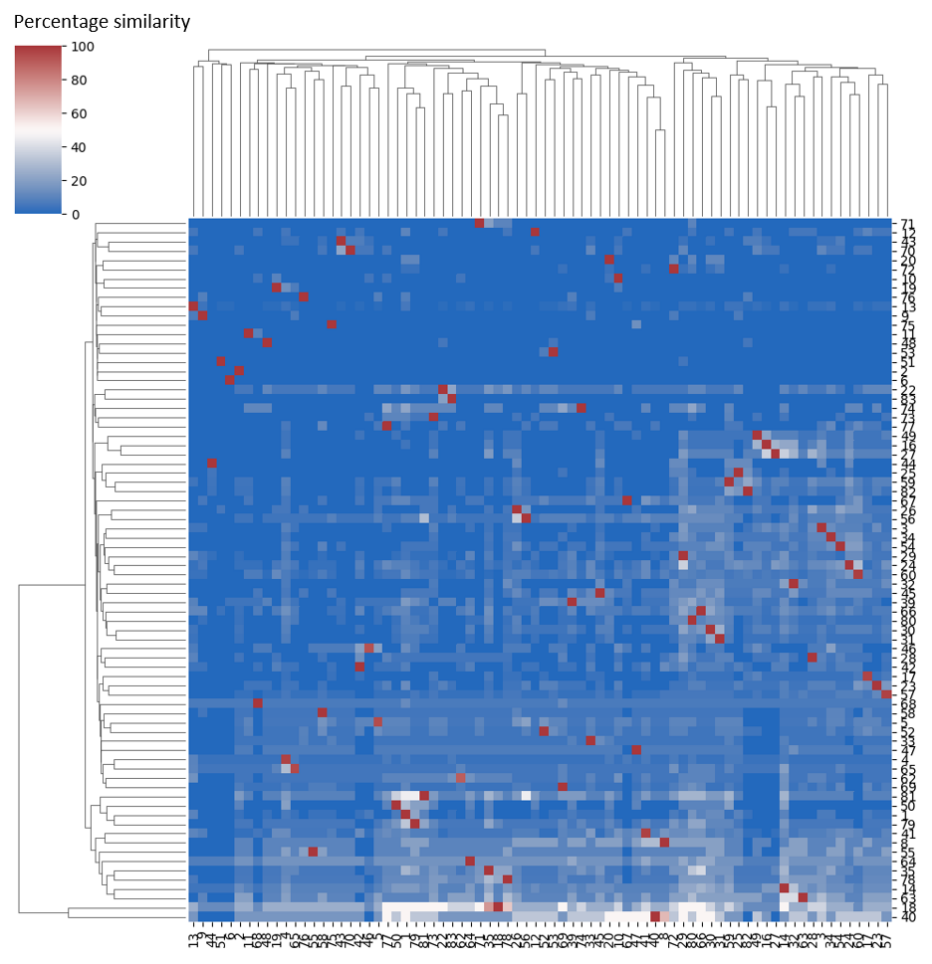
Similarity between theories based on percentage of construct mentions. Theories are paired, with each row and each column representing a theory denoted by number (see Extended data for theory numbers; osf.io/dcqft ( Hale *et al.*, 2020)). Rows show the percentage of constructs from the numbered theory ‘mentioned in’ other theories. Columns show the percentage of constructs from other theories that the numbered theory ‘mentions’. Dark blue indicates low similarity in constructs. Each theory is 100% contained in / by itself (dark red cells). Rows and columns in the matrix are ordered by hierarchical clustering using the average of the Euclidean distances, such that rows and columns that have more similar values to each other are grouped closer together. The clustering is shown in the dendrograms to the left and top of the figure.

One of the most promising applications of the theory database is to ask how theories themselves are related. While there are potentially many ways that a measure of theory similarity can be computed for the OBMS formalism, and we plan to explore these further in future work, an initial naïve metric could be based on the construct mentions as calculated on a per-construct basis above, generalised to a per-theory basis. Using this approach, we calculated for each theory the percentage of its constructs that are mentioned in another theory. Doing this for all possible pairwise combinations of theories in the dataset, we constructed a heat map showing the degree to which each pair of theories contain similar constructs ( Figure 6).

The value in each cell of the heat map is the percentage of the row theory’s constructs, which are also mentioned in the column theory. All percentages are calculated with respect to the row theory, numbered along the right side of the matrix (see *Extended data* for theory numbers; osf.io/dcqft ( Hale *et al.*, 2020)). Thus, every pairwise combination of theories has two values in the heat map. For example, we can see that the percentage of constructs from theory 40 mentioned in theory 8 (light pink cell) is higher than the percentage of constructs from theory 8 mentioned in theory 40 (light blue cell).

For a given theory, the heat map can be interpreted by row or by column. Rows show the percentage of constructs from the numbered theory that are ‘mentioned in’ other theories. For example, a row of red cells would indicate 100% of constructs from the numbered theory are *mentioned in* every other theory. Rows with high percentage values are more likely if the row theory has few constructs. Columns show the percentage of constructs from other theories that the numbered theory ‘mentions’. For example, a column of red cells would indicate the numbered theory *mentions* 100% of the constructs from another theory for all theories in the matrix. Columns with high percentage values are more likely if the column theory has many constructs. The rows and columns in the matrix are ordered by hierarchical clustering using the average of the Euclidean distances, such that rows and columns that have more similar values to each other are grouped closer together. The clustering is shown in the dendrograms to the left and top of Figure 6.

Two groupings of lighter blue rows towards the bottom of the matrix contain theories with higher percentages of their constructs mentioned across other theories. The bottom-most grouping contains theories 40 and 18 (“Motivation-Opportunities-Abilities” and “Focus Theory of Normative Conduct” respectively), while other rows that show higher percentages of mentions include theories 8, 55, 64 and 63 among others (“COM-B”, “Risk as Feelings Theory”, “Social Consensus Model” and “Social Cognitive Theory” respectively). This can be considered evidence of the generality of these theories according to the percentage similarity metric, which may indicate theories that describe a broader part of the domain of behaviour change or include commonly used construct names.

Column-wise, there are several groupings of theories that appear to mention higher percentages of constructs from other theories that are not all widely-mentioned overall; for example, theories 72, 29, 80, 66, 30 and 31 form one such group (“Systems model of behaviour change”, “I-Change model”, “Theory of triadic influence”, “Social ecological model of behaviour change”, “Information-Motivation-Behavioural Skills Model” and “Information-Motivation-Behavioural Skills Model of Adherence Behaviour”). We can expect to see such groupings as a result of theories covering a similar aspect or part of the overall behaviour change domain. On the other hand, the left-most group of dark blue columns indicates a group of theories that mention few constructs from other theories, which may be expected from theories that are somewhat dissimilar to most of the domain. This includes theories 13, 9, 44, 51, and 6 (namely, the “Diffusion of Innovations, “Consumption as social practices”, “Precaution Adoption Process Model”, the “Rational addiction model”, and “Change theory”). Inspection of their respective constructs verifies that indeed, most of these theories are using different terminologies or construct names than the more typical language used in the bulk of the theories in the behaviour change domain. For example, “Change theory” appears in this grouping, which includes construct names such as “Freezing”, “Restraining forces” and the “Quasi-stationary equilibrium” that are not shared with any other theories.

## Discussion

For 71 theories included in the present study, a diagram using the OBMS could be generated which adequately captured the constructs and relationships proposed in the theory. Pooling these diagrams with five previously generated diagrams using a nearly identical methodology ( West *et al.*, 2019), we derived a database of formal representations of each theory in the form of construct-relationship-construct triples.

Our findings demonstrate that the OBMS could be scaled up to represent a large proportion of existing theories of behaviour change. Of 83 theories identified in a previous systematic review ( Davis *et al.*, 2015), 76 are now represented in our database in a common systematic format. To capture this range of theories, only one additional relationship type, “Transitions to”, was needed in addition to the thirteen types of relationship identified in the development of the OBMS by West *et al.* (2019). The initial thirteen relationship types were grouped by West *et al.* (2019) into three basic types: causal (e.g. “influences”), semantic (e.g. “type of”) and structural (e.g. “part of”). “Transitions to”, on the other hand, describes a process relationship whereby one state changes to another. Our findings suggest this type of relationship was not uncommonly used across theories, but only accounts for a small fraction of the overall number of propositions made.

Our initial analyses on the theory database give a picture of the landscape of existing behaviour change theories. The theories themselves vary greatly in size, with a correlation between number of constructs per theory and number of relationships between constructs. Unsurprisingly, “behaviour” was the most commonly referenced construct across theories, but, perhaps surprisingly, a fifth of theories did not use the word behaviour at all. Instead, they used other terms for behaviour, such as “action” (e.g. Transtheoretical Model; Prochaska & DiClemente, 1982) and “action or inaction response” (Norm Activation Theory; Schwartz, 1975). This illustrates a practical consideration for representing and comparing theories systematically: construct names alone provide limited information about the actual construct. Additional information such as the definition of that construct and its ontological relationship to others is needed to search and compare across theories accurately. Without this information, it is hard to be precise about commonalities and gaps that may exist among theories. Our next steps (outlined below) will address this.

Our findings also indicated some groups of overlapping theories. On the one hand, we could identify groups of theories that contained constructs commonly used across the whole dataset, which could be interpreted as more general theories in terms of describing a broad part of the behaviour change domain or using commonly mentioned construct names. These were typically less detailed theories, containing fewer than the average number of constructs and triples. One of the theories in this grouping, Social Cognitive Theory ( Bandura, 1986), was also identified in previous network analysis as contributing to 12 of the 83 theories in the corpus, based on explicit indication in the theory sources ( Michie *et al.*, 2014). However, the others from the grouping contributed to three or fewer theories in the network analysis, suggesting there is not necessarily high overlap between theories on the construct similarity metric used in the present study and the contribution metric used in the previous study.

On the other hand, we could also identify groups of theories that mention similar constructs from other theories which are not necessarily widely mentioned overall, thus representing a similar part of the overall domain, as well as theories which mentioned very few constructs contained in other theories. Furthermore, we have shown that formalising theories into the OBMS triple format can allow for exploration of the ‘neighbourhood network’ of all the propositions made about how what influences or is influenced by a given construct such as beliefs or attitudes relates to other constructs across the whole dataset. These findings demonstrate potential for the OBMS to facilitate synthesis and identify ‘canonical’ theories, which we will discuss in the next steps.

Despite the apparent groupings of similar theories, we found that only around 20% of construct names from the 76 theories appear in more than one theory, and this is further evidenced by the dominance of low percentages of mentions between theories. This is a striking result, considering that all of the theories pertained to behaviour change, and therefore more commonalities might be expected within a domain. It highlights the extent of theoretical heterogeneity in the labels used to describe different phenomena across the field, and further illustrates the need to map the constructs that have been used in theories to shared ontologies, so that overlaps in construct definitions could also be identified.

As well as facilitating generation of the theory database, the OBMS provides a tool which researchers can use to formally specify theories under development. Because the OBMS is ontologically-based, it is able to accommodate a variety of different relationship types, including causal, semantic, structural and process relationships, and could accommodate more types of propositions if needed. This means the OBMS is compatible with, but not restricted to, quantitative approaches to theory specification such as mathematical equations or simulation models, recommended in methodologies for psychological theory construction and testing (e.g. Borsboom *et al.*, 2020; Forstmann *et al.*, 2011; Oberauer & Lewandowsky, 2019). Using the OBMS as an integrative semantic framework built on the existing database could help to shift from current practice, characterised by small group ‘ownership’ of theories and a climate which discourages theory development during early career stages ( Borsboom *et al.*, 2020; van Rooij & Baggio, 2020), to a more open and collaborative approach within and between teams.

A current limitation of the OBMS format is its representation of constructs which relate to a relationship, such as a moderator. Because the triple format only allows for construct-relationship-construct propositions, in some cases we needed to treat relationships as constructs in order for triples with relationships as targets to be expressible. This type of workaround is commonly called ‘reification’ in the computer science and conceptual modelling literature. In the OBMS format it results in triples such as “Feedback-influences-[the 'Goals' to 'Persistence' Influences (*) relationship]” (Goal Setting Theory; Locke, 1968; Locke & Latham, 2002). This creates additional complexity when comparing and combining theories, because triples can be the targets of other triples, thus can be “nested”. However, the ability to capture relationships such as moderators, which influence other relationships, is essential for a faithful representation of the semantic content of some theories. In the future, we may explore the use of a more expressive formalism than the basic construct-relationship-construct triple format in order to capture these elements of theories more intuitively.

The OBMS database structure can be searched and used as a basis for mathematical and logical inference. While some preliminary results are illustrated in this manuscript, to make more powerful inferences it will be necessary to assign an unambiguous meaning to the constructs. We plan to address this in the next steps of the project by annotating the constructs in the theories to ontology entities from e.g. the Behaviour Change Intervention Ontology ( Michie *et al.*, 2020b). Ontology annotations of this type provide additional metadata, such as synonyms and definitions, and also a formal logical semantics in the relevant ontology language (e.g. OWL). This will allow theories to be checked for logical consistency internally and in combination with other theories. It will then be possible to determine which theories are mutually inconsistent (i.e. cannot both be true) based on logical contradictions across the subsets of annotated ontology entities. This added functionality will mean that in future steps we can build a ‘canonical’ theory that is most consistent with the combined theoretical representation across all the theories. It will also allow researchers to identify and assess different predictions made by theories from the same domain.

## Conclusions

The ontology-based modelling system (OBMS) is a systematic approach that can be used to represent a large body of behaviour change theories. Feedback from theory authors suggests that this method captures theory propositions adequately, with nearly half of theory authors requiring no revisions of the initial OBMS representation of their theory. This study extended the initial development of the system ( West *et al.*, 2019), and a significant output of the research is a database of 76 published behaviour change theories which can be used for comparison and synthesis. Initial analyses on the database indicate this body of theories is characterised by heterogeneous construct names, as well as some areas of overlap. This suggests that greater integration and synthesis of theories is possible and would be beneficial. The next steps of the research programme will facilitate this through mapping the theory database to ontologies of behaviour change and using computational methods to synthesise theories.

## Data availability

### Underlying data

Open Science Framework: An ontology-based modelling system (OBMS) for representing behaviour change theories, https://doi.org/10.17605/OSF.IO/4DVPE ( Hale *et al.*, 2020)

This project contains the following underlying data:

- Feedback and revision data for 69 theory diagrams sent to authors or experts.csv (Raw data on type of feedback received, category of changes made, and theories finalised at each round of the feedback and revision process; osf.io/6pw5y)
- Theories [OSF Storage sub-folder] (Folder of CSV data files for 76 theories represented in OBMS format, exported from LucidChart and post-processed for the identification of construct-relationship-construct triples; osf.io/pfe84/files)

### Extended data

Open Science Framework: An ontology-based modelling system (OBMS) for representing behaviour change theories, https://doi.org/10.17605/OSF.IO/4DVPE ( Hale *et al.*, 2020)

This project contains the following extended data:

- List of numbered theories screened for inclusion from Michie *et al.* (2014).csv ( osf.io/dcqft)
- Guide to the visual representation of relationship types as sent to theory authors.pdf ( osf.io/8fvdb)
- Finalised theory diagrams for 76 theories represented using the OBMS [Folder of PDF files] ( osf.io/4urjc/files)

Data are available under the terms of the Creative Commons Attribution 4.0 International license (CC-BY 4.0).

## Software availability

The searchable database of theories that have been represented using the Ontology-Based Modelling System (OBMS) is available at: https://humanbehaviourchange.org/theory-database

Open Science Framework: An ontology-based modelling system (OBMS) for representing behaviour change theories, https://doi.org/10.17605/OSF.IO/4DVPE ( Hale *et al.*, 2020)

- TheoryDatabase.py (Source code scripted in Python to parse CSV data from 76 theories represented in OBMS format, in order to create a database of triples; osf.io/d7hzy)
- GenerateTheoryStatistics.py (Source code scripted in Python to conduct statistical analyses on the database of 76 theories, as presented in the Results section, Figure 3– Figure 6 and Table 2; osf.io/hx4mw)

Source code is available under the terms of the GNU General Public License (GPL) 3.0.

## References

[ref-1] Ajzen I: The theory of planned behavior. *Organ Behav Hum Dec.* 1991;50(2):179–211. 10.1016/0749-5978(91)90020-T

[ref-2] Bagozzi RP: The Self-Regulation of Attitudes, Intentions, and Behavior. *Soc Psychol Quart.* 1992;55(2):178–204. 10.2307/2786945

[ref-3] Bandura A: Social foundation of thought and action: A social-cognitive view. Englewood Cliffs.1986 Reference Source

[ref-4] Borland R: Understanding hard to maintain behaviour change: A dual process approach.xi, 239 Wiley-Blackwell.2014 10.1002/9781118572894

[ref-5] Borsboom D van der Maas H Dalege J: Theory Construction Methodology: A practical framework for theory formation in psychology.[Preprint]. *PsyArXiv.* 2020 10.31234/osf.io/w5tp8 33593167

[ref-6] Carey RN Connell LE Johnston M: Behavior Change Techniques and Their Mechanisms of Action: A Synthesis of Links Described in Published Intervention Literature. *Ann Behav Med.* 2019;53(8):693–707. 10.1093/abm/kay078 30304386 PMC6636886

[ref-7] Connell LE Carey RN de Bruin M: Links Between Behavior Change Techniques and Mechanisms of Action: An Expert Consensus Study. *Ann Behav Med.* 2019;53(8):708–720. 10.1093/abm/kay082 30452535 PMC6636885

[ref-8] Craig P Dieppe P Macintyre S: Developing and evaluating complex interventions: The new Medical Research Council guidance. *BMJ.* 2008;337:a1655. 10.1136/bmj.a1655 18824488 PMC2769032

[ref-9] Davis R Campbell R Hildon Z: Theories of behaviour and behaviour change across the social and behavioural sciences: A scoping review. *Health Psychol Rev.* 2015;9(3):323–344. 10.1080/17437199.2014.941722 25104107 PMC4566873

[ref-10] DiClemente CC Prochaska JO Fairhurst SK: The process of smoking cessation: An analysis of precontemplation, contemplation, and preparation stages of change. *J Consult Clin Psychol.* 1991;59(2):295–304. 10.1037//0022-006x.59.2.295 2030191

[ref-11] Ellson J Gansner ER Koutsofios E: Graphviz and dynagraph - static and dynamic graph drawing tools. *Graph Drawing Software.* 2003;127–148. 10.1007/978-3-642-18638-7_6

[ref-12] Fisher JD Fisher WA: Changing AIDS-risk behavior. *Psychol Bull.* 1992;111(3):455–474. 10.1037/0033-2909.111.3.455 1594721

[ref-13] Flay BR d’Avernas J Best A: Cigarette Smoking: Why Young People Do It and Ways of Preventing It. In: *Pediatric and Adolescent Behavioral Medicine* 1983;132–183. Reference Source

[ref-14] Flay BR Petraitis J: The Theory of Triadic Influence: A New Theory of Health Behavior With Implications for Preventive Interventions. *Adv Med Sociol.* 1994;4:19–44. Reference Source

[ref-15] Flay BR: On improving the chances of mass media health promotion programs causing meaningful changes in behavior. In: M. Meyer (Ed.), *Health education by television and radio* 1981;56–91. Saur. Reference Source

[ref-16] Flay BR DiTecco D Schlegel RP: Mass Media in Health Promotion: An Analysis Using an Extended Information-Processing Model. *Health Educ Q.* 1980;7(2):127–147. 10.1177/109019818000700203 7275639

[ref-17] Forstmann BU Wagenmakers EJ Eichele T: Reciprocal relations between cognitive neuroscience and formal cognitive models: Opposites attract? *Trends Cogn Sci.* 2011;15(6):272–279. 10.1016/j.tics.2011.04.002 21612972 PMC3384559

[ref-18] Fried EI: Lack of theory building and testing impedes progress in the factor and network literature.2020 10.31234/osf.io/zg84s

[ref-20] Gourlan M Bernard P Bortolon C: Efficacy of theory-based interventions to promote physical activity. A meta-analysis of randomised controlled trials. *Health Psychol Rev.* 2016;10(1):50–66. 10.1080/17437199.2014.981777 25402606

[ref-21] Guest O Martin AE: How computational modeling can force theory building in psychological science.2020 10.31234/osf.io/rybh9 33482070

[ref-22] Hale J Hastings J West R: An ontology-based modelling system (OBMS) for representing behaviour change theories applied to 76 theories.2020 10.17605/OSF.IO/4DVPE PMC7653641 33215048

[ref-23] Haslbeck JMB Ryan O Robinaugh D: Modeling Psychopathology: From Data Models to Formal Theories. [Preprint]. *PsyArXiv.* 2019 10.31234/osf.io/jgm7f PMC10259162 34735175

[ref-24] Johnston M Carey RN Connell Bohlen LE: Linking behavior change techniques and mechanisms of action: Triangulation of findings from literature synthesis and expert consensus. [Preprint]. *PsyArXiv.* 2018 10.31234/osf.io/ur6kz PMC8158171 32749460

[ref-25] Locke EA: Toward a theory of task motivation and incentives. *Organ Behav Hum Perform.* 1968;3(2):157–189. 10.1016/0030-5073(68)90004-4

[ref-26] Locke E Latham G: Building a Practically Useful Theory of Goal Setting and Task Motivation. A 35-Year Odyssey. *Am Psychol.* 2002;57(9):705–717. 10.1037//0003-066x.57.9.705 12237980

[ref-27] Michie S Johnston M Abraham C: Making psychological theory useful for implementing evidence based practice: A consensus approach. *Qual Saf Health Care.* 2005;14(1):26–33. 10.1136/qshc.2004.011155 15692000 PMC1743963

[ref-28] Michie S Prestwich A: Are interventions theory-based? Development of a theory coding scheme. *Health Psychol.* 2010;29(1):1–8. 10.1037/a0016939 20063930

[ref-29] Michie S Richardson M Johnston M: The Behavior Change Technique Taxonomy (v1) of 93 Hierarchically Clustered Techniques: Building an International Consensus for the Reporting of Behavior Change Interventions. *Ann Behav Med.* 2013;46(1):81–95. 10.1007/s12160-013-9486-6 23512568

[ref-30] Michie S Thomas J Mac Aonghusa P: The Human Behaviour-Change Project: An artificial intelligence system to answer questions about changing behaviour [version 1; peer review: not peer reviewed]. *Wellcome Open Res.* 2020a;5:122. 10.12688/wellcomeopenres.15900.1 32566761 PMC7287511

[ref-31] Michie S West R Campbell R: ABC of Behaviour Change Theories. Silverback Publishing.2014 Reference Source

[ref-32] Michie S West R Finnerty AN: Representation of behaviour change interventions and their evaluation: Development of the Upper Level of the Behaviour Change Intervention Ontology [version 1; peer review: awaiting peer review]. *Wellcome Open Res.* 2020b;5:123 10.12688/wellcomeopenres.15902.1 PMC7868854 33614976

[ref-33] Muthukrishna M Henrich J: A problem in theory. *Nat Hum Behav.* 2019;3(3):221–229. 10.1038/s41562-018-0522-1 30953018

[ref-34] Norris E Finnerty AN Hastings J: A scoping review of ontologies related to human behaviour change. *Nat Hum Behav.* 2019;3(2):164–172. 10.1038/s41562-018-0511-4 30944444

[ref-35] Oberauer K Lewandowsky S: Addressing the theory crisis in psychology. *Psychon Bull Rev.* 2019;26(5):1596–1618. 10.3758/s13423-019-01645-2 31515732

[ref-36] Painter JE Borba CPC Hynes M: The Use of Theory in Health Behavior Research from 2000 to 2005: A Systematic Review. *Ann Behav Med.* 2008;35(3):358–362. 10.1007/s12160-008-9042-y 18633685

[ref-37] Pavlov IP: Conditioned reflexes: An investigation of the physiological activity of the cerebral cortex. Oxford University Press.1927;430 Reference Source 10.5214/ans.0972-7531.1017309 PMC4116985 25205891

[ref-38] Prestwich A Sniehotta FF Whittington C: Does theory influence the effectiveness of health behavior interventions? Meta-analysis. *Health Psychol.* 2014;33(5):465–474. 10.1037/a0032853 23730717

[ref-39] Prochaska JO DiClemente CC: Transtheoretical therapy: Toward a more integrative model of change. *Psycho Theory Res Prac.* 1982;19(3):276–288. 10.1037/h0088437

[ref-40] Rosenstock IM: The Health Belief Model and Preventive Health Behavior. *Health Edu Mono.* 1974;2(4):354–386. 10.1177/109019817400200405

[ref-41] Schwartz S: The Justice of Need and the Activation of Humanitarian Norms. *J Soc Issue.* 1975;31(3):111–136. 10.1111/j.1540-4560.1975.tb00999.x

[ref-42] van Rooij I Baggio G: Theory before the test: How to build high-verisimilitude explanatory theories in psychological science. [Preprint]. PsyArXiv.2020 10.31234/osf.io/7qbpr PMC8273840 33404356

[ref-43] Weber M: Economy and Society: An Outline of Interpretive Sociology. University of California Press.1978 Reference Source

[ref-44] West R Brown J: Theory of Addiction. John Wiley & Sons.2013.

[ref-45] West R Godinho CA Bohlen LC: Development of a formal system for representing behaviour-change theories. *Nat Hum Behav.* 2019;3(5):526–536. 10.1038/s41562-019-0561-2 30962614

[ref-19] Wolfenden L Goldman S Stacey FG: Strategies to improve the implementation of workplace-based policies or practices targeting tobacco, alcohol, diet, physical activity and obesity. *Cochrane Database Syst Rev.* 2018;11(11):CD012439. 10.1002/14651858.CD012439.pub2 30480770 PMC6362433

