# Supplementary material for: An ontology-based modelling system (OBMS) for representing behaviour change theories applied to 76 theories
Source: Wellcome Open Res. 2020 Jul 23;5:177. [Version 1] doi: 10.12688/wellcomeopenres.16121.1 (PMC7653641; doi:10.12688/wellcomeopenres.16121.1)
Supplement: Peer review history

# Peer review: Reviewer response for version 1

**DOI:** 10.21956/wellcomeopenres.17695.r40646

**Published:** 2020-11-09

**Authors:** Austin S. Baldwin

**Affiliations:** 1 Department of Psychology, Southern Methodist University, Dallas, TX, USA

In this paper, the authors report on the creation and development of a searchable database (an ontology-based modeling system [OBMS]) of behavior change theories and their related constructs and relationships. The database includes 76 different behavior change theories that went through a rigorous identification and coding process prior to inclusion (71 of the theories identified and coded in the phase of the project reported here). The creation, development, and validation of these tools are a very important and needed undertaking to systematically synthesize the vast array of behavior change theories. The database has the potential to be a critical tool in facilitating scientific advances in behavior change theory and interventions. This paper is well-constructed and well-written. The rigorous process for identifying and coding the various theories, constructs, and relationships included feedback from theory authors and is an important strength of the paper. Several of the tables and figures are also helpful in conveying what types of information are available in the database and potential uses. Finally, the inclusion of database materials relevant to this paper on an open repository (OSF) is an important strength of the paper and the project. I support the indexing of this paper.

 I also have a few suggestions for the authors that largely center on more clearly discussing the implications and elaborating on potential uses of the database. For example, the authors might consider including concrete examples of how researchers could use the database to design interventions and test theories. I think addressing the following issues would strengthen an already strong paper:

1. It is not entirely clear how researchers might use information available in the database to inform intervention design (e.g., Which theory should we use? Does it matter which we use?), or to properly design interventions to test a theory and inform its refinement. Are there concrete examples of how researchers might approach these questions using the database? Is the database ultimately agnostic to these issues? Either way, it would be helpful to clarify for readers.
2. The information about overlap in constructs among theories conveyed in the heat map (Figure 6) is quite interesting. One persistent challenge in advancing behavior change theory is in the redundancies that exists across various theories that creates barriers to building more cumulative literatures. How do the authors envision this information and database functionality could potentially be used? For example, could the information conveyed in the heat map be used to winnow or refine redundant theories? Elaborating on potential uses of this information would be helpful.
3. Using construct labels as they are labeled in the theories rather than using conceptual definitions of the constructs likely contributed to the unexpected lack of overlap in constructs across theories (20%). This approach almost certainly underestimates the degree of conceptual overlap across theories (see Noar & Zimmerman, 2005, Health Education Research, for consideration of this issue). The first reviewer had a similar comment, and I agree with the first reviewer that more clearly and directly discussing this issue would be helpful.
4. I think it would be helpful to clarify the theoretical implications and practical utility of the “network neighborhood” of a construct conveyed in Figure 5.

Is the work clearly and accurately presented and does it cite the current literature?

Yes

If applicable, is the statistical analysis and its interpretation appropriate?

Not applicable

Are all the source data underlying the results available to ensure full reproducibility?

Yes

Is the study design appropriate and is the work technically sound?

Yes

Are the conclusions drawn adequately supported by the results?

Yes

Are sufficient details of methods and analysis provided to allow replication by others?

Yes

Reviewer Expertise:

Health psychology, health behavior change

I confirm that I have read this submission and believe that I have an appropriate level of expertise to confirm that it is of an acceptable scientific standard.

# Peer review: Reviewer response for version 1

**DOI:** 10.21956/wellcomeopenres.17695.r40344

**Published:** 2020-09-28

**Authors:** Diane Dixon

**Affiliations:** 1 Institute of Applied Health Sciences, School of Medicine, Medical Sciences and Nutrition, University of Aberdeen, Aberdeen, UK

The preponderance of theories is a significant barrier to the development of a cumulative evidence base for understanding and changing human behaviour. This study is part of a programme of work that is developing the tools to address the issue of theory proliferation and construct overlap. I would strongly support its indexing.

 ‘What’s in a name?’, asked Juliet and whilst I’d generally avoid challenging Shakespeare it turns out the answer is ‘quite a lot, really’. As the authors discuss in the introduction, behavioural science is not bounded by the discipline imposed by the properties of matter that benefits the natural sciences. Our reliance on natural language poses challenges to behavioural science because it is unbounded. I see the work described in the paper as part of a wider attempt to develop tools and mechanisms that will enable behavioural medicine to identify the boundaries and shared content of the theories and constructs it regularly, and not so regularly uses. This is a worthwhile endeavour.

 I have some comments to share with the authors:

- They are collating constructs by collating construct names and partial names. This generates figure 4. However, not all of the ‘constructs’ displayed in figure 4 would generally be recognised as ‘constructs’. For example, whilst self-efficacy is a recognised construct ‘social’ and ‘personal’ are not ‘constructs’ in the generally accepted meaning of the term, e.g. ‘social support’, ‘social norm’ are very different constructs. I am not arguing against the authors’ method here rather that I am struggling with their use of the term ‘construct’ to label the groupings that are produced by the process of collation – ‘all constructs are equal but some are more equal than others’…some aren’t even constructs.
- The authors themselves are surprised by the finding that even for partial matching only 20% of constructs appear in more than one theory. Either they have a few theories that contain a very large number of unique constructs or there is a problem with the use of non-standardised terms to label constructs. I am struggling to believe the 20% figure is a true representation of reality rather than an artefact of poor construct labelling. The authors allude to this in the discussion, but I wonder if they might consider strengthening the discussion of this possibility.
- The included theories all seem to lean towards conceptualising behaviour as a result of deliberative processes. Perhaps I am in error here, but if not, it would be useful if the authors could consider how environmental models of behaviour, especially behaviourist approaches, might be accommodated within the OBMS.
- Similarly, how well does the OBMS accommodate important sociodemographic, cultural and socially constructed influences on behaviour? These are often treated as moderators of belief-behaviour relationships. The authors point out the limitations of the reification approach to accommodating moderators and I would very much encourage the authors to think about how moderating factors can be better represented in the OBMS and/or whether the OBMS provides an opportunity to consider possible non-mediating mechanisms by which sociodemographic and cultural factors act to shape behaviour.

Is the work clearly and accurately presented and does it cite the current literature?

Yes

If applicable, is the statistical analysis and its interpretation appropriate?

Not applicable

Are all the source data underlying the results available to ensure full reproducibility?

Yes

Is the study design appropriate and is the work technically sound?

Yes

Are the conclusions drawn adequately supported by the results?

Yes

Are sufficient details of methods and analysis provided to allow replication by others?

Yes

Reviewer Expertise:

health psychology, behavioural science

I confirm that I have read this submission and believe that I have an appropriate level of expertise to confirm that it is of an acceptable scientific standard.
